# Optimal design and nonlinear dynamic characteristics of titanium /steel drill pipe composite drill string for ultra-deep drilling

**DOI:** 10.1038/s41598-023-47156-y

**Published:** 2023-11-22

**Authors:** Qiang Liu, Bo Zhou, Feng Chen, Ning Li, Junfeng Xie, Mifeng Zhao, Qinfeng Di, Chun Feng, Shengyin Song, Chengxian Yin

**Affiliations:** 1 State Key Laboratory for Performance and Structural Safety of Petroleum Tubular Goods and Equipment Materials, CNPC Tubular Goods Research Institute, No. 89, Jinye 2nd Road, Xi’an, 710077 Shaanxi China; 2 Petroleum Engineering Institute, Tarim Oil Field Company of CNPC, Kuerle, 841000 Xinjiang China; 3 School of Mechatronics Engineering and Automation, Shanghai University, Shanghai, 200072 China; 4 Shanghai Institute of Applied Mathematics and Mechanics, School of Mechanics and Engineering Science, Shanghai University, Shanghai, 200072 China

**Keywords:** Mechanical engineering, Mathematics and computing

## Abstract

Titanium drill pipe have promising application prospects in ultra-deep drilling, but the nonlinear dynamic characteristics of composite drill strings are very complex. It is very important to use titanium drill pipe safely, economically, and effectively in the drilling process. In this paper, different schemes of the titanium/steel drill pipe composite drill string was designed, and the statics and dynamics characteristics of these composite drill strings were analyzed. The design scheme of titanium/steel composite drill strings were optimized by using whirling characteristics, dynamic stress and vibration acceleration. The results show that the titanium drill pipe can effectively improve the tensile allowance of the wellhead and reduce the whirling velocity, dynamic stress and vibration acceleration of the drill string. By comparing and analyzing the dynamic characteristics of 3 kinds of composite drill strings, the use of titanium drill pipe for the lower 2000 m of the 4′′ drill pipe have the smaller dynamic stress and lateral vibration, and less collision with the wellbore. Based on the analysis of vibration and dynamic characteristics, an optimal scheme of drilling operation parameters for the titanium/steel composite drill string was formed finally, which can provide guidance for the use of the drill string composed of titanium/steel drill pipe in natural gas drilling of ultra-deep well.

## Introduction

With the exploration and exploitation of oil and gas around the world continue to go deep into the unconventional environment such as high-temperature and high-pressure (HTHP) conditions, ultra-deep wells, extended reach wells, multi-fishbone horizontal wells and off-shore exploitation, higher requirements are put forward for the performance of oil country tubular goods (OCTG)^1,2^. The development of ultra-deep wells over 7000 m in China is mainly concentrated in the northwest region, accounting for 55% of the total number of development wells. These ultra-deep wells have bottomhole temperature as high as 170–190 °C, some even reaching 200 °C, and the wellhead pressure is as high as 110–130 MPa. Moreover, these wells are rich in highly corrosive media such as Cl^−1^ and CO_2_, and have high bottomhole pressure. Coupled with the poor physical properties of the reservoir, the exploration and exploitation of these ultra-deep wells are very difficult^3,4^. Researchers have conducted extensive research on various difficulties encountered during the drilling process, achieving good research results and promoting the progress of drilling technology^5–9^. Under such ultra-deep, HTHP working conditions, drill strings bears complex loads such as internal/external pressure, tension/compression, wear, rotational fatigue, bending and temperature stress, which leads to long drilling cycle, frequent accidents and high costs in ultra-deep wells. This has become a serious constraint on the exploration and exploitation of deep oil and gas resources.

Titanium alloy has become an attractive candidate material for OCTG and off-shore components in harsh service condition, owing to its high specific strength, low density (only 56% of steel), low elastic modulus (only 52% of steel), excellent corrosion resistance, long fatigue life and outstanding mechanical properties^10–12^. As early as the late twentieth century, the United States took the lead in developing titanium drill pipe products, and used titanium drill pipes in Texas and the Gulf of Mexico for deep well offshore conditions and ultra-short radius horizontal wells^13–15^. Field application shows that the use of titanium drill pipe for extended reach drilling operations can effectively reduce the hook load by 30%, the torque by 30–40%, and the effective drilling depth can be increased by 5000ft. Moreover, the supporting mobile drilling rig can realize rapid drilling from the well, saving up to 30% of investment^16,17^. Titanium alloy materials can withstand a high temperature of 260 °C and have excellent corrosion resistance^18^. With a 25% reduction in titanium drill pipe wall thickness, the safety factor of the drill string is 3.5 times that of the chromium steel/nickel-based drill strings^19,20^.

With the increasing demand for drilling in deep and ultra-deep wells, scholars over the world have also conducted research on the drilling capabilities and applicability of titanium drill pipe in recent years. Zhanghua Lian et al.^21^ established a theoretical model of drill string dynamics in gas drilling of horizontal wells, and found that the contact force between wellbore and drill string was large and helical buckling of drill string can be caused without the lubrication and damping effects of drilling fluid in gas drilling. Wanying Liu and Carsten Blawert^22^ formed ceramic coatings on the surface of titanium drill pipe by plasma electrolytic oxidation (PEO) technology and proved that the coating showed extremely significant wear resistance due to the physical barrier of graphene nano-sheets. Xiaohua Zhu et al.^23^ established a theoretical full-scale titanium drill pipe dynamics model based on the Hamilton principle, and analyzed the ability of titanium drill pipe to reduce friction, resistance to deformation, and axial force transmission and its influence factor. Kang Hongbing et al.^24^ studied the applicability of titanium drill pipe in work over operations in ultra-deep wells, and conducted tensile check and hydraulic performance calculations on titanium/steel composite drill string.

At present, titanium drill pipes are mainly used in short radius horizontal wells, and there are few reports on their application in ultra-deep wells. The main reason for this is that the drilling string in ultra-deep wells faces extreme load environments such as huge axial force, large torque, high temperature and high pressure. The complex dynamic characteristics of the drilling string pose serious challenges to its safety. In ultra-deep drilling over 8000 m, titanium drill pipes is too expensive for the entire drill string, therefore, the use of titanium/steel drill pipe composite drill string become a more feasible solution. The use of composite drill string design can not only effectively reduce the weight of the drill string for ultra-deep wells, but also optimize the dynamic characteristics of the composite drill string, thereby greatly reducing the probability of drill string failure, which is considered to be the most competitive technology for ultra-deep wells and deep-sea drilling. Unfortunately, there are few researches on the design of titanium/steel drill pipe composite drill string and the performance of the composite drill string. For composite drill strings, the length and location of titanium drill pipes have a significant impact on the whirling and vibration characteristics of the drill string. The mechanism of the influence of flexible titanium drill pipes on the dynamic characteristics of the composite drill string is not clear, and the selection of the length, location, and drilling operation parameters of titanium drill pipes in the composite drill string lacks theoretical basis. In this paper, 4′′ titanium drill pipe and steel drill pipe are used to form 3 kinds of titanium/steel composite drill strings, the statics and dynamics model of these titanium/steel composite drill strings were established. The node iteration method is used to judge whether contact occurs, and the Newmark method is used to calculate the spatial configuration of the drill-string. Based on the stress, whirling, and vibration characteristics of the composite drill string, the length and location of use of titanium drill pipes in the composite drill string of ultra-deep wells were determined. On this basis, the dynamic characteristics of the titanium/steel drill pipe composite drill string were analyzed based on different WOB and rotational speed conditions, an optimal scheme of operating parameters for the composite drill string is formed final. This paper can provide important technical guidance for the use of titanium/steel drill pipe composite drill string and drilling safety control in the drilling of ultra-deep well and deep-sea drilling.

## Theoretical model

Since the drill string will be subjected to complex loads such as tension, compression, bending, torsion and vibration in ultra-deep drilling, it is not clear whether the drill string, especially the composite drill string, can meet the performance requirements under these conditions. Both statics and dynamics characteristics of the composite drill string should be studied.

Due to the drill string has the characteristics of extremely large slenderness ratio, the assumptions in the finite element model can be made as the following:

1. The mass distribution of the drill string is uniform and isotropic.
2. The drill string is elastic, and the deformation of the drill string is small.
3. Ignored the stress caused by temperature effect on the drill string.
4. Ignored the stiffness and local dimensional features of threaded connections.
5. The cross-sectional area of the drill string will not be warped.

**Figure 1:**
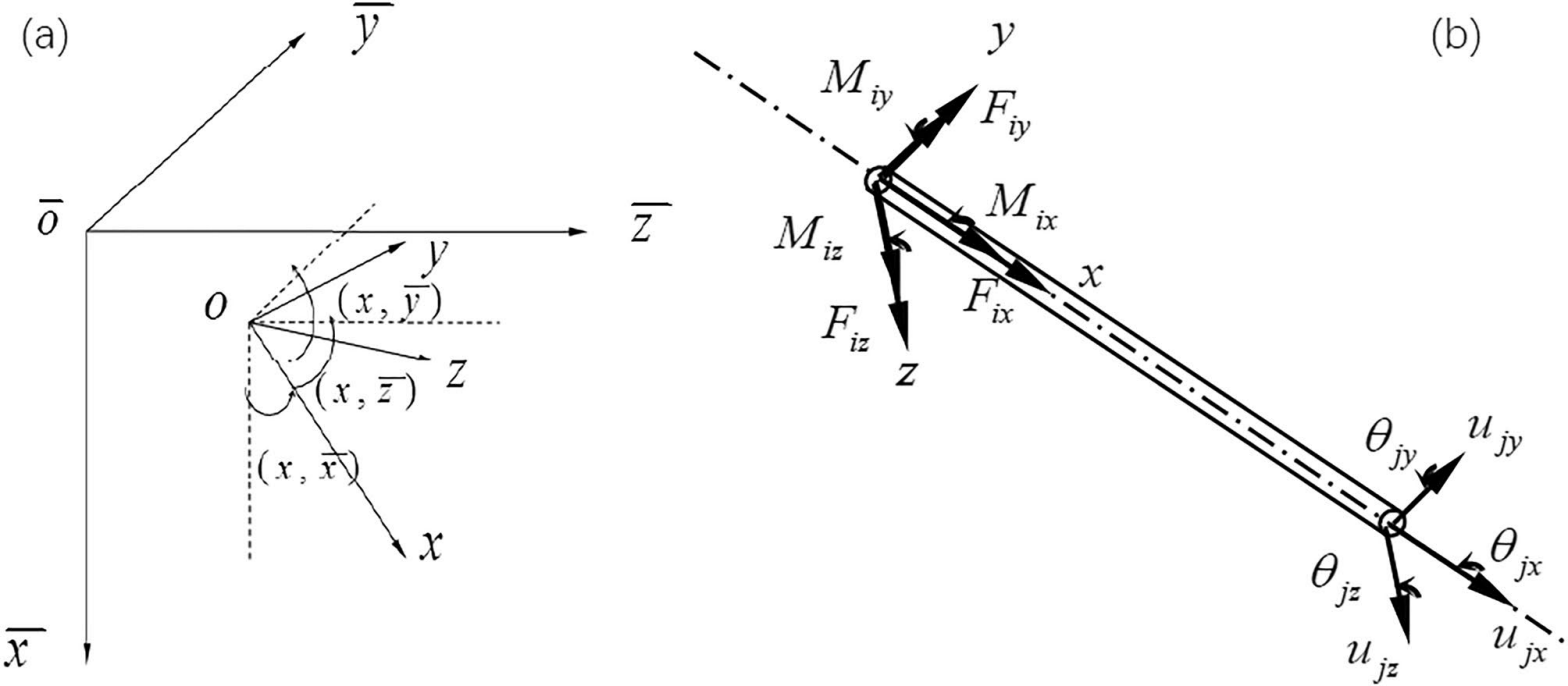
Established analysis coordinate system (**a**) and spatial beam elements (**b**).

The analysis coordinate system is established with the wellhead $\overline{o}$ as the origin, as shown in Fig. 1a, the ${ }\overline{x }$ axis is along the gravity direction, the $\overline{y}, \overline{z}$ axis is pointing to the north and east, respectively. $o - xyz$ is the local coordinate system of the element fixed on the drill string.

Euler–Bernoulli 3D beam elements has been used, and the element of drill string is simplified shown as in Fig. 1b, ($F_{ix} , F_{iy} , F_{iz}$) are the loads in the three directions of the node *i*, ($M_{ix} , M_{iy} , M_{iz}$) are the moments in the three directions of the node *i*; ($u_{jx} , u_{jy} , u_{jz}$) are the displacement in each three directions of the node *j*, ($\theta_{jx} , \theta_{jy} , \theta_{jz}$) is the torsion angle of node *j* in three directions.

The inclination angle of the node *i* is $\varphi_{i}$, and the azimuth angle is $\psi_{i}$. Through coordinate conversion, the coordinate conversion relationship between the global coordinate system and the local coordinate system of the beam element can be obtained and showed as following:

1 $$\lambda_{i} = \left[ {\begin{array}{*{20}c} {l_{{x\overline{x}}} } & {l_{{x\overline{y}}} } & {l_{{x\overline{z}}} } \\ {l_{{y\overline{x}}} } & {l_{{y\overline{y}}} } & {l_{{y\overline{z}}} } \\ {l_{{z\overline{x}}} } & {l_{{z\overline{y}}} } & {l_{{z\overline{z}}} } \\ \end{array} } \right] = \left[ {\begin{array}{*{20}c} {cos\varphi_{i} } & {sin\varphi_{i} cos\psi_{i} } & {sin\varphi_{i} sin\psi_{i} } \\ { - sin\varphi_{i} } & {cos\varphi_{i} cos\psi_{i} } & {cos\varphi_{i} sin\psi_{i} } \\ 0 & { - sin\psi_{i} } & {cos\psi_{i} } \\ \end{array} } \right]$$

where, $l_{{x\overline{x}}}$, $l_{{x\overline{y}}}$, $l_{{x\overline{z}}} { }$ in the formula (1) are the direction cosines of the local coordinate *x* to the global coordinate system $\overline{x},{ }\overline{y},{ }\overline{z}{ }$. Similarly, $l_{{y\overline{x}}}$, $l_{{y\overline{y}}}$, $l_{{y\overline{z}}} , l_{{z\overline{x}}}$, $l_{{z\overline{y}}}$, $l_{{z\overline{z}}}$ are the direction cosines of the local coordinates *y* and *z* to the global coordinates, respectively.

### Finite element model

The whole drill string is discretized into finite elements, the 3D finite element model of the titanium/steel composite drill string is established. Due to the irregularity of the wellbore and the variable cross-section of the drilling tool, the gap between the drill string and wellbore changes constantly. There is frequent and uncertain contact between the drill string and the wellbore, which significantly affects the dynamic characteristics of the drill string. The mechanical characteristics of drill string is actually a dynamic problem including geometric nonlinearity and contact nonlinearity^25^. The drill string dynamics finite element model in this paper is based on the Hamilton principle.

The Hamilton principle in dynamic analysis of drill string can be expressed as follows: in all possible movements of drill string from the state at moment *a* to the state at moment *b* under the condition that the displacement boundary is satisfied, the real movement of drill string makes Hamilton functional take the extremum. When the drill string moves in the wellbore filled with viscous drilling fluid, it will rub and collide with the wellbore. The viscous damping force exerted by the drilling fluid and the dynamic friction force exerted by the wellbore on the drill string are non conservative forces. Therefore, when applying Hamilton principle to the drill string system, energy dissipation caused by non conservative forces should be considered, which can be obtained as follows:

2 $${\varvec{\delta}}\int_{{t_{1} }}^{{t_{2} }} {\left( {T - V} \right)} \text{d}t + {\varvec{\delta}}\int_{{t_{1} }}^{{t_{2} }} W \text{d}t = 0$$

where ${\varvec{\delta}}$ is the variational symbol; $T$ is the kinetic energy of the drill string, J; $V$ is the potential energy of drill string, J; $W$ is the total work done by the conservative force and non conservative force on the drill string, J.

The generalized nodal coordinates of any element in drill string can be defined as:

3 $${\varvec{u}}^{e} = \left[ {u_{ix} , u_{iy} , u_{iz} , \theta_{ix} , \theta_{iy} , \theta_{iz} , u_{jx} , u_{jy} , u_{jz } ,\theta_{jx} , \theta_{jy} , \theta_{jz} } \right]$$

Since kinetic energy $T$ is a function of velocity and potential energy $V$ is only a function of displacement, kinetic energy and potential energy can be expressed as:

4 $$T = \frac{1}{2}\int_{0}^{{l_{e} }} {\rho \left\{ {A\left[ {\left( {\dot{u}_{x} } \right)^{2} + \left( {\dot{u}_{y} } \right)^{2} + \left( {\dot{u}_{z} } \right)^{2} } \right] + I_{x} \left( {\dot{\theta }_{x}^{2} + 2\dot{\theta }_{x} \dot{\theta }_{y} \theta_{z} } \right) + I_{yz} \left( {\dot{\theta }_{y}^{2} + \dot{\theta }_{z}^{2} } \right)} \right\}} {\varvec{d}}x$$

5 $$V = \frac{1}{2}\int\limits_{v} {{\varvec{\sigma}}^{T} } {\varvec{\varepsilon}}\text{d}{\text{v}}$$

According to the basic assumption mentioned above, the material of the drill string is elastic, so the stress–strain relationship of the drill string satisfies the generalized Hooke's law, namely:

6 $${{\varvec{\upsigma}}} = {\mathbf{D\varepsilon }}\quad$$

where ${\mathbf{D}}$ is the elastic matrix.

7 $${\mathbf{D}} = \frac{E(1 - \upsilon )}{{(1 + \upsilon )(1 - 2\upsilon )}}\left[ {\begin{array}{*{20}l} 1 \hfill & {\frac{\upsilon }{1 - \upsilon }} \hfill & {\frac{\upsilon }{1 - \upsilon }} \hfill & 0 \hfill & 0 \hfill & 0 \hfill \\ {\frac{\upsilon }{1 - \upsilon }} \hfill & 1 \hfill & {\frac{\upsilon }{1 - \upsilon }} \hfill & 0 \hfill & 0 \hfill & 0 \hfill \\ {\frac{\upsilon }{1 - \upsilon }} \hfill & {\frac{\upsilon }{1 - \upsilon }} \hfill & 1 \hfill & 0 \hfill & 0 \hfill & 0 \hfill \\ 0 \hfill & 0 \hfill & 0 \hfill & {\frac{1 - 2\upsilon }{{2(1 - \upsilon )}}} \hfill & 0 \hfill & 0 \hfill \\ 0 \hfill & 0 \hfill & 0 \hfill & 0 \hfill & {\frac{1 - 2\upsilon }{{2(1 - \upsilon )}}} \hfill & 0 \hfill \\ 0 \hfill & 0 \hfill & 0 \hfill & 0 \hfill & 0 \hfill & {\frac{1 - 2\upsilon }{{2(1 - \upsilon )}}} \hfill \\ \end{array} } \right]$$

where $E$ and $\upsilon$ are the Young's modulus and Poisson's ratio of the material, respectively.

For drill strings with a large slenderness ratio, it is usually assumed that the stress contributing to the corresponding strain energy is the stress $\sigma_{xx}$ and $\tau_{xy}$ on the cross-section perpendicular to the axis of the beam element. Based on this:

8 $$\sigma_{yy} = \sigma_{zz} = \tau_{yz} = 0$$

By substituting the above equation into the generalized Hooke's law Eq. (6), the following equation can be obtained:

9 $$\begin{gathered} \varepsilon_{yy} = \varepsilon_{zz} = - \upsilon \varepsilon_{xx} \hfill \\ \gamma_{yz} = 0 \hfill \\ \end{gathered}$$

Substituting Eqs. (8) and (9) into Eq. (5), the potential energy expression can be obtained as follows:

10 $$V = \frac{1}{2}\int\limits_{v} {\left[ {E\varepsilon_{xx}^{2} + G\left( {\gamma_{xy}^{2} + \gamma_{yz}^{2} } \right)} \right]} \text{d}v$$

Due to the significant axial and torsional deformation of the drill string under the action of gravity and torque, Green strain is adopted, namely:

11 $$\begin{aligned} \varepsilon_{xx} & = \frac{{\partial u_{x} }}{\partial x} + \frac{1}{2}\left[ {\left( {\frac{{\partial u_{x} }}{\partial x}} \right)^{2} + \left( {\frac{{\partial u_{y} }}{\partial x}} \right)^{2} + \left( {\frac{{\partial u_{z} }}{\partial x}} \right)^{2} } \right] \\ \gamma_{xy} & = \frac{{\partial u_{x} }}{\partial y} + \frac{{\partial u_{y} }}{\partial x} + \frac{{\partial u_{x} }}{\partial x}\frac{{\partial u_{x} }}{\partial y} + \frac{{\partial u_{y} }}{\partial x}\frac{{\partial u_{y} }}{\partial y} + \frac{{\partial u_{z} }}{\partial x}\frac{{\partial u_{z} }}{\partial y} \\ \gamma_{xz} & = \frac{{\partial u_{x} }}{\partial z} + \frac{{\partial u_{z} }}{\partial x} + \frac{{\partial u_{x} }}{\partial x}\frac{{\partial u_{x} }}{\partial z} + \frac{{\partial u_{y} }}{\partial x}\frac{{\partial u_{y} }}{\partial z} + \frac{{\partial u_{z} }}{\partial x}\frac{{\partial u_{z} }}{\partial z} \\ \end{aligned}$$

Assuming that the translational and rotational displacement of the drill string under axial force, torque, and bending moment are independent, the displacement at any point on the cross-section of the beam element can be expressed as:

12 $$\begin{aligned} u_{x} & = u_{xo} - y\theta_{z} + z\theta_{y} \\ u_{y} & = u_{yo} - z\theta_{x} \\ u_{z} & = u_{zo} + y\theta_{x} \\ \end{aligned}$$

where $y$ and $z$ are the horizontal coordinates of any point on the cross-section of the drill string in the local coordinate system.

According to the cross-sectional assumption, the axis displacement $u_{xo} , \, u_{yo} , \, u_{zo}$ and cross-sectional (torsion) angle $\theta_{x} , \, \theta_{y} , \, \theta_{z}$ in Eq. (12) are only functions of the local coordinate $x$. In addition, due to neglecting the shear deformation of the drill string, the relationship between the deflection and rotation angle can be obtained:

13 $$\theta_{y} = - \frac{{\partial u_{{{\text{zo}}}} }}{\partial x}{, }\theta_{z} = \frac{{\partial u_{yo} }}{\partial x}$$

Substituting Eqs. (11)–(13) into Eqs. (10), ignoring higher-order sub terms, the following can be obtained:

14 $$\begin{aligned} V & = \frac{EA}{2}\int_{0}^{{l_{e} }} {\left( {\frac{{\partial u_{{\text{o}}} }}{\partial x}} \right)}^{2} {\varvec{d}}x + \frac{{GI_{x} }}{2}\int_{0}^{{l_{e} }} {\left( {\frac{{\partial \theta_{x} }}{\partial x}} \right)}^{2} {\varvec{d}}x + \frac{{EI_{yz} }}{2}\int_{0}^{{l_{e} }} {\left( {\frac{{\partial \theta_{z} }}{\partial x}} \right)}^{2} {\varvec{d}}x \\ & \;\;\;\; + \frac{{EI_{yz} }}{2}\int_{0}^{{l_{e} }} {\left( {\frac{{\partial \theta_{y} }}{\partial x}} \right)}^{2} {\varvec{d}}x + \frac{EA}{2}\int_{0}^{{l_{e} }} {\left( {\frac{{\partial u_{o} }}{\partial x}} \right)}^{3} {\varvec{d}}x + \frac{EA}{2}\int_{0}^{{l_{e} }} {\frac{{\partial u_{o} }}{\partial x}\left[ {\left( {\theta_{y} } \right)^{{2}} { + }\left( {\theta_{{\text{z}}} } \right)^{{2}} } \right]} {\varvec{d}}x \\ & \;\;\;\; + \frac{{3EI_{yz} }}{2}\int_{0}^{{l_{e} }} {\frac{{\partial u_{o} }}{\partial x}\left[ {\left( {\frac{{\partial \theta_{y} }}{\partial x}} \right)^{{2}} { + }\left( {\frac{{\partial \theta_{z} }}{\partial x}} \right)^{{2}} } \right]} {d}x + \frac{{EI_{x} }}{2}\int_{0}^{{l_{e} }} {\frac{{\partial u_{o} }}{\partial x}} \left( {\frac{{\partial \theta_{x} }}{\partial x}} \right)^{2} {\varvec{d}}x \\ & \;\;\;\; + \frac{{\left( {E - G} \right)I_{x} }}{2}\int_{0}^{{l_{e} }} {\frac{{\partial \theta_{x} }}{\partial x}} \left( {\theta_{y} \frac{{\partial \theta_{z} }}{\partial x} - \theta_{z} \frac{{\partial \theta_{y} }}{\partial x}} \right){\varvec{d}}x \\ \end{aligned}$$

Substitute the expressions of the kinetic energy, potential energy and external force terms of the beam element into the Lagrange equation, the finite element equation of drill string dynamics can be derived:

15 $${\varvec{M}}^{{\varvec{e}}} \ddot{U}^{e} + {\varvec{C}}^{{\varvec{e}}} \dot{U}^{e} + {\varvec{K}}^{{\varvec{e}}} U^{e} = {\varvec{F}}^{{\varvec{e}}}$$

where $\ddot{U}^{e} , \dot{U}^{e} , U^{e}$ are the generalized acceleration, generalized velocity and generalized displacement vector of the element respectively, ${\varvec{F}}^{{\varvec{e}}}$ Sis the generalized force vector, ${\varvec{M}}^{{\varvec{e}}} ,\user2{ C}^{{\varvec{e}}} ,\user2{ K}^{{\varvec{e}}}$ are the element mass matrix, damping matrix and stiffness matrix, respectively.

The boundary conditions of the drill string are: the upper end of the drill string (i.e. the wellhead) is hinged at the center of the wellbore, the axial direction is pulled by the hook load, and a specific rotational speed is applied in the torsion direction. The lateral motion at the lower end of the drill string (i.e. at the drill bit) is also a hinge boundary condition, while the axial and torsional motions are respectively affected by the WOB and torque, and their magnitude depends on the interaction with the bit and the rock.

For PDC bits, the expressions for torque and WOB at the bit can be expressed as^26^:

16 $$W_{b} = W_{0} + W_{{\text{a}}} {\sin}\left( {n\tilde{\theta }_{x1} } \right)$$

17 $$T_{b} = \mu_{s} W_{b} r_{b}$$

where $W_{{\text{b}}}$ is the WOB on the bit, N; $W_{{0}}$ is the static WOB on the bit, N; $W_{{\text{a}}}$ is the fluctuation amplitude of WOB on the bit, N; $\tilde{\theta }_{x1}$ is the torsional displacement of the bit, rad; $T_{b}$ is the torque on the bit, N.m; $\mu_{s}$ is the static friction coefficient; ${\text{r}}_{b}$ is the radius of the bit, m.

In addition, when the radial displacement of the drill string is greater than the gap between the drill string and the wellbore, the movement of the drill string will also be constrained by the wellbore. In this case, the force exerted by the wellbore on the drill string includes radial contact force, tangential friction force and torque.

**Figure 2:**
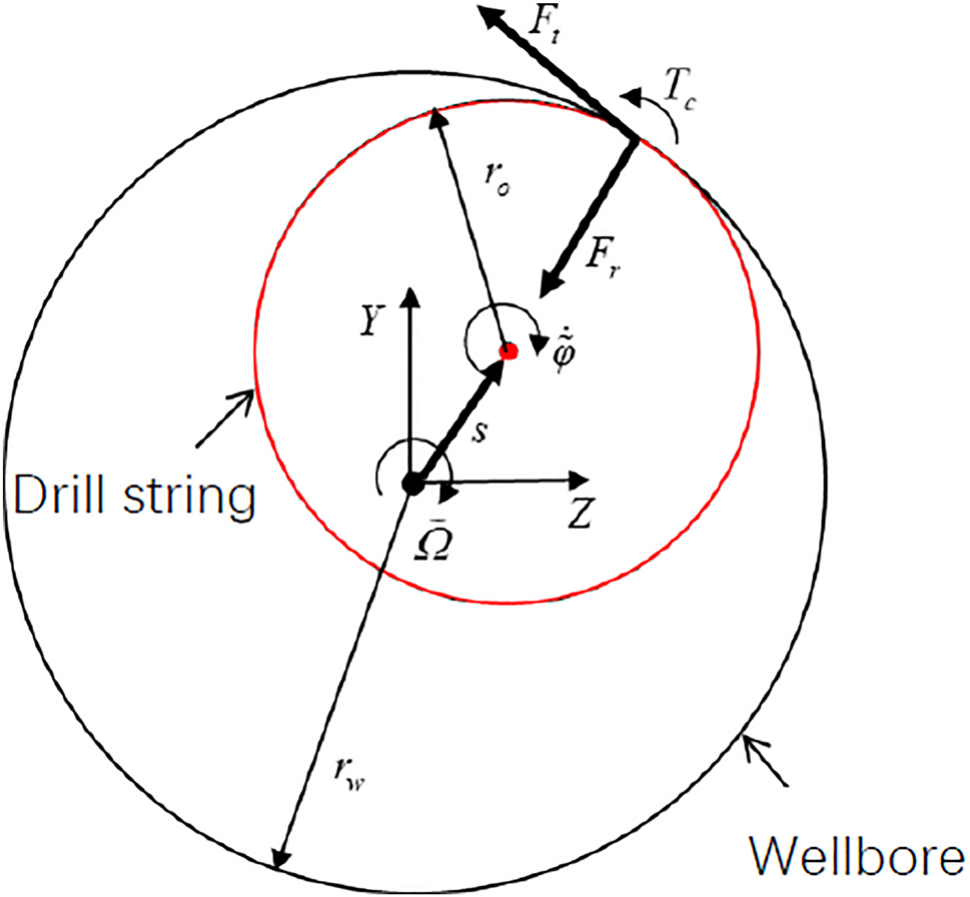
Schematic diagram of the contact model between drill string and wellbore.

The contact model between the drill string and the wellbore is established by the Hertz contact theory, as shown in Fig. 2, The radial contact force *F*_*r*_, tangential friction force *F*_*t*_ and friction torque *T*_*c*_ can be expressed as:

18 $$\begin{aligned} F_{{\text{r}}} & { = }\left\{ {\begin{array}{*{20}l} { - K_{h} \left( {s + r_{o} - r_{w} } \right)^{\frac{3}{2}} } \hfill & {s \ge r_{w} - r_{o} } \hfill \\ 0 \hfill & {s < r_{w} - r_{o} } \hfill \\ \end{array} } \right. \\ F_{t} & = - \mu_{b} F_{r} \text{sign}\left( {\tilde{\Omega }} \right) \\ T_{c} & = F_{t} {\text{r}}_{o} \\ \end{aligned}$$

where ${\text{s}} = \sqrt {u_{yo}^{2} + u_{zo}^{2} }$ is the radial displacement of the drill string, m, *K*_*h*_ is the wellbore stiffness, N m^-3/2^, $\mu_{b}$ is the kinetic friction coefficient between the drill string and the wellbore, $\tilde{\Omega }$ is the relative rotational velocity between the drill string and the wellbore, rad/s.

For the solution of dynamic model of drill string, the node iteration method is used for spatial discretization, and the Newmark-β method is used for temporal discretization. If the difference between the initial displacement and the actual displacement is large, the node iteration method needs many iterations to calculate the actual displacement, which seriously affects the calculation efficiency. The global stiffness matrix method is accurate and fast, but in dealing with nonlinear problems, due to the large scale of the matrix of the ultra-deep drill string model, the global stiffness matrix method needs to carry out multiple large-scale matrix operations, which takes a long time to solve, and may even be unable to calculate. Therefore, combined with the advantages of the node iteration method and the global stiffness matrix method, the global stiffness matrix method is used to preliminarily solve the model without considering the contact firstly, and the displacement obtained is taken as the initial displacement, then the node iteration method is used to solve the problem with considering the contact nonlinearity^27^. In this way, the solving time of the dynamic model of the drill string in ultra-deep wells can be greatly reduced.

### Wellbore trajectory description

**Figure 3:**
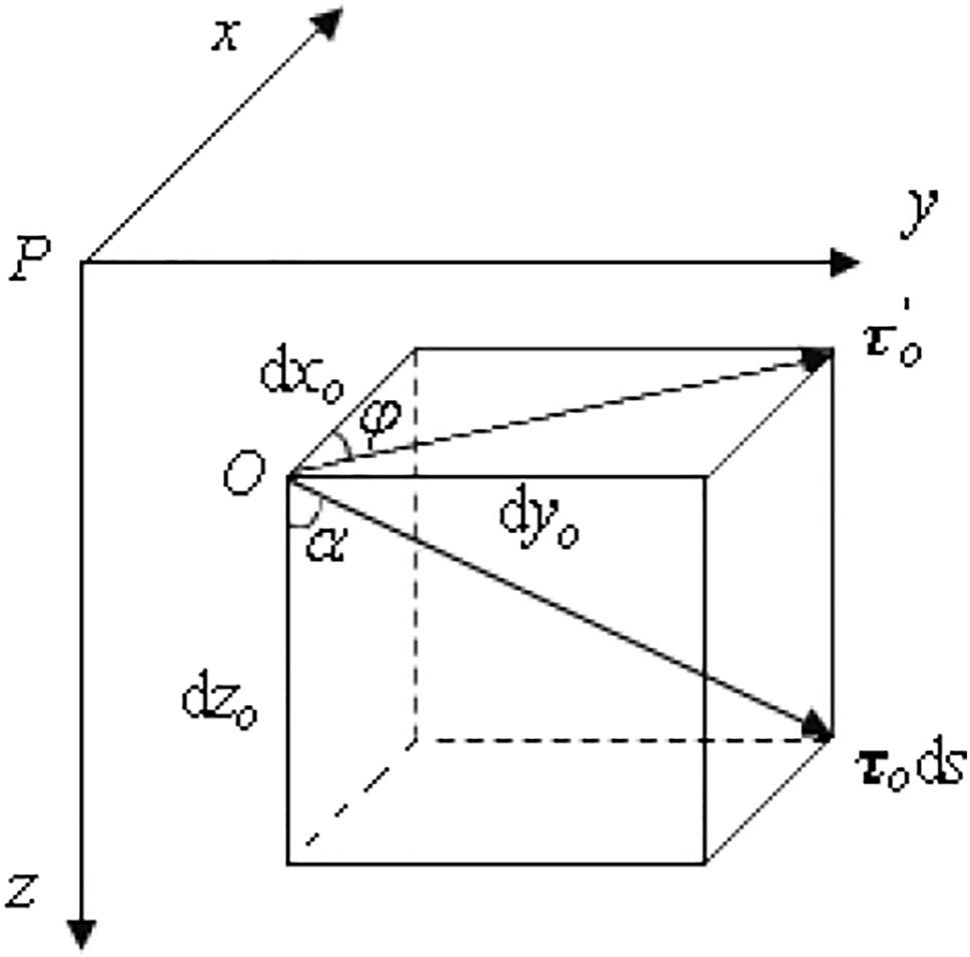
Schematic diagram of geometric relationship of 3D curved well trajectory.

In order to more realistically calculate the actual drilling process in the field, it is necessary to describe the wellbore according to the actual measured wellbore trajectory. Cartesian coordinate system and natural coordinate system were used to describing the spatial characteristics of 3D curved well segments, as shown in Fig. 3. Taking the wellhead *P* as the origin of the coordinate system, *i*, *j* and *k* are the unit vectors representing *x*, *y* and *z* along the coordinate axes in the Cartesian coordinate system, respectively.

The geometrical position of any section on the wellbore axis in three-dimensional space can be described by the vector radius *r*_*0*_:

19 $${\varvec{r}}_{o} \left( s \right) = x_{o} \left( s \right){\varvec{i}} + y_{o} \left( s \right){\varvec{j}} + z_{o} \left( s \right){\varvec{k}}$$

20 $${\text{d}}{\varvec{r}}_{{\text{o}}} = {\text{d}}x_{o} \left( s \right){\varvec{i}} + {\text{d}}y_{o} \left( s \right){\varvec{j}} + {\text{d}}z_{o} \left( s \right){\varvec{k}} = {\varvec{\tau}}_{o} {\text{d}}s$$

where ***τ***_*o*_ is the unit vector in the tangential direction along the trajectory of the wellbore axis; the angle *α* between ***τ***_*o*_ and ***k*** defined as the inclination angle, rad; the angle φ between the projection of ***τ***_*o*_ on the ***P***_***xy***_ plane ${\varvec{\tau}}_{o}{\prime}$ and ***i*** is called the azimuth angle, rad; ***s*** is the arc length of the wellbore, m.

After differential geometry calculation and simplification, the curvature $k_{0}$ and the torsion $T_{0}$ of the wellbore trajectory curve can be expressed as:

21 $$k_{o}^{2} = \left( {\frac{{{\text{d}}\alpha }}{{{\text{d}}s}}} \right)^{{2}} + {\text{sin}}^{{2}} \alpha \left( {\frac{{{\text{d}}\varphi }}{{{\text{d}}s}}} \right)^{{2}}$$

22 $$T_{o} = \frac{1}{{k_{o}^{2} }}\left\{ {\left( {\frac{{{\text{d}}\alpha }}{{{\text{d}}s}}\frac{{{\text{d}}^{{2}} \varphi }}{{{\text{d}}s^{2} }} - \frac{{{\text{d}}\varphi }}{{{\text{d}}s}}\frac{{{\text{d}}^{{2}} \alpha }}{{{\text{d}}s^{{2}} }}} \right){\text{sin}}\alpha + \left[ {{2}\frac{{{\text{d}}\varphi }}{{{\text{d}}s}}\left( {\frac{{{\text{d}}\alpha }}{{{\text{d}}s}}} \right)^{{2}} + {\text{sin}}^{{2}} \alpha \left( {\frac{{{\text{d}}\varphi }}{{{\text{d}}s}}} \right)^{{3}} } \right]{\text{cos}}\alpha } \right\}$$

**Figure 4:**
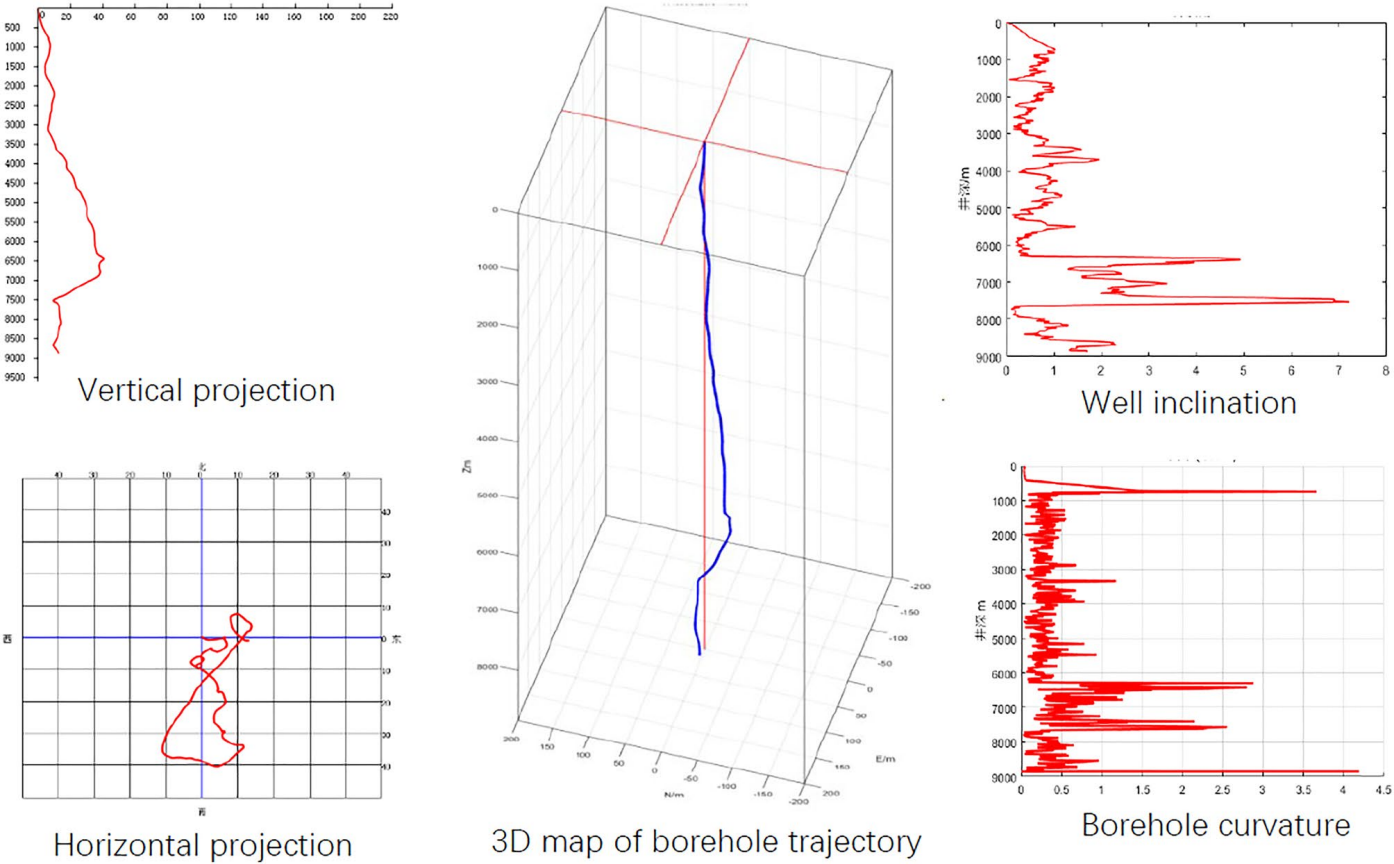
Well trajectory characteristics.

The radius of curvature method is used to interpolate the wellbore trajectory measurement data of an ultra-deep well in western China^24^, as shown in Table 1 and Fig. 4. The overall angle change rate (also known as dogleg severity or wellbore curvature) in Table 1 refers to the angular change of the wellbore axis per unit wellbore length in 3D space. In drilling engineering, the coordinate system usually uses north and east coordinates, represented by N and E, which refer to the displacement value of the measurement position in the horizontal coordinate system with the wellhead as the coordinate origin. The positive sign in the east–west coordinates represents the east, while the negative sign represents the west. The positive sign in the north–south coordinates represents the south, and the negative sign represents the north. The curvature radius method assumes that the wellbore trajectory is circular in both vertical and horizontal projections, and the constructed wellbore trajectory is relatively smooth and consistent with the actual situation. The closure distance refers to the horizontal distance from the measurement position on the horizontal projection surface to the wellhead, while the closure azimuth refers to the angle on the horizontal projection surface that rotates clockwise from the north direction to the line connecting the measurement position and the wellhead. It can be seen from the figure that the inclination angle of the well is relatively large in the 6000–8000 m section, the maximum inclination angle is 7.21°, and the curvature reaches 4.4°/30 m.

**Table 1:** Wellbore trajectory measurement data.

Well depth (m)	Vertical depth (m)	Inclination angle (°)	Azimuth angle (°)	Overall angle change rate (°/25 m)	East–west coordinate (m)	North–south coordinate (m)	Closure Azimuth (°)	Closure distance (m)
0.00	0.00	0.00	0.00	0.00	0.00	0.00	0.00	0.00
126.30	126.30	0.20	111.84	0.07	0.15	0.10	55.92	0.19
418.30	418.29	0.50	118.44	0.03	1.77	− 0.65	110.25	1.89
725.65	725.62	1.02	50.44	0.10	5.59	− 0.28	92.87	5.60
1017.65	1017.59	0.78	221.10	0.09	6.30	− 3.00	115.45	6.97
1517.65	1517.56	0.26	95.76	0.37	2.51	− 3.21	142.01	4.07
2017.65	2017.51	0.68	189.35	0.05	− 0.22	− 8.23	181.52	8.23
2517.65	2517.50	0.21	59.85	0.20	− 0.78	− 8.05	185.51	8.08
3017.65	3017.49	0.40	309.08	0.10	0.07	− 6.08	179.32	6.08
3517.65	3517.41	1.04	112.80	0.43	− 0.07	− 10.11	180.39	10.11
4017.65	4017.31	0.42	57.72	0.38	4.74	− 17.05	164.48	17.70
4517.65	4517.26	0.81	231.35	0.17	4.64	− 22.31	168.25	22.79
5017.65	5017.20	0.48	143.02	0.46	5.10	− 28.75	169.93	29.20
5517.65	5517.17	1.35	126.02	0.14	6.45	− 32.03	168.61	32.67
6017.65	6017.14	0.19	106.75	0.29	10.45	− 33.61	162.73	35.20
6517.65	6516.64	2.97	291.08	1.62	− 0.05	− 38.60	180.07	38.60
7017.65	7016.30	3.27	21.86	0.40	− 9.85	− 30.04	198.15	31.61
7517.65	7515.17	7.13	36.26	0.44	7.31	− 5.32	126.03	9.04
8017.65	8014.73	0.70	24.10	0.31	13.63	3.28	76.48	14.02
8517.65	8514.67	0.72	244.30	0.21	8.48	6.86	51.03	10.90

The interval of actual measurement data is 20 m. Limited to space, only some data are listed here.

## Structure design and mechanical characteristic analysis of titanium/steel drill pipe composite drill string

The cost of titanium drill pipes is expensive, and using titanium drill pipes for the entire drill strings requires high costs. In ultra-deep drilling applications, it is necessary to use a drill string design that combines steel drill pipes with titanium drill pipes. It is very important to determine the appropriate length of titanium drill pipes in the drill string design to control drilling costs. Moreover, the stiffness of titanium drill pipes is relatively small, and the dynamic characteristics of the titanium/steel drill pipe composite drill string are significantly different from conventional steel drill strings. Improper use may exacerbate the vibration of the drill string and increase the risk of drill string failure. Based on the static and dynamic models of the drill string described in “Theoretical model” section, this study analyzed the stress characteristics of 3 kinds of composite drill strings using MATLAB 2020b software, and compared them with the stress characteristics of conventional steel drill strings. A titanium/steel drill pipe composite drill string design scheme was determined, and its drilling operation parameters were optimized.

### Structure design

**Figure 5:**
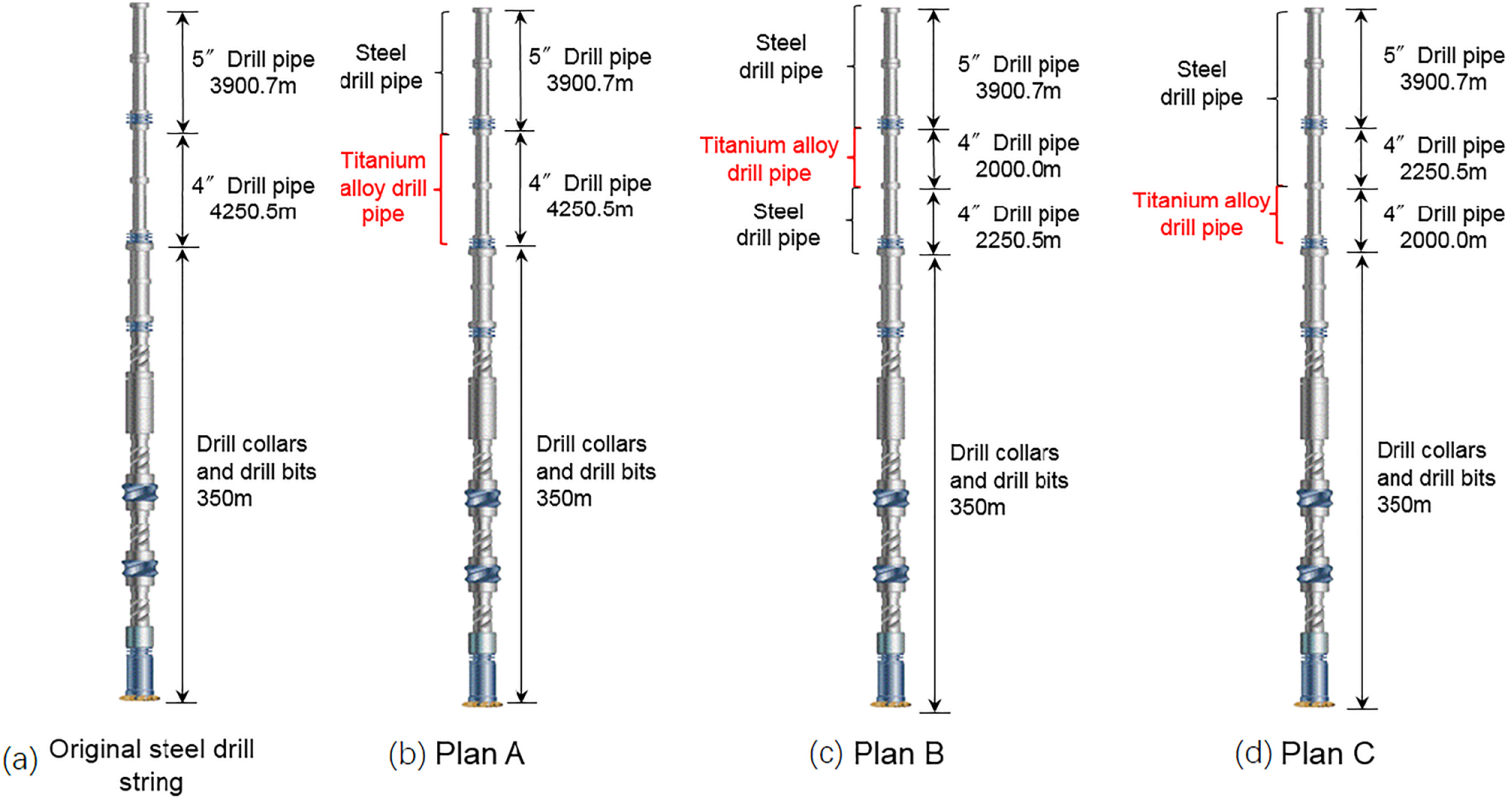
Schematic diagram of original steel drill string (**a**) and three titanium/steel drill pipe composite drill string plans (**b**)–(**d**).

Based on the actual wellbore structure of an ultra-deep well (over 8500m) in western China, 3 kinds of titanium/steel drill pipe composite drill string structure were designed, as shown in Fig. 5a. The maximum axial tensile force of the original steel drill string is 2020 kN and located at the wellhead (the density of the drilling fluid used in the calculation was 1.45 g/cm^3^). The tensile strength of the S135 grade 5′′ steel drill pipe is 3311 kN, then the tensile allowance of the wellhead drill pipe is only 960 kN considering the requirement of a safety factor of 0.9. In order to further improve the safety during drilling and the ability to deal with emergencies, a section of titanium drill pipe used to reduce the weight of the drill string. Considering that the weight of 4′′ steel drill pipe is 23.8 kg/m, while the weight of 4′′ titanium drill pipe is only 14.8 kg/m, 2000 m of 4′′ steel drill pipe replaced with 4′′ titanium drill pipe can increase the tensile allowance of the wellhead drill pipe to 1130 kN.

Considering drilling safety and cost, 3 kinds of titanium/steel drill pipe composite drill string were designed, they are the plan A of using titanium drill pipes for all the 4′′ drill pipes, the plan B of using titanium drill pipes for the upper 2000 m of the 4′′ drill pipes, and the plan C of using titanium drill pipe for the lower 2000 m of the 4′′ drill pipes, as shown in Fig. 5b–d.

### Static characteristics analysis

**Figure 6:**
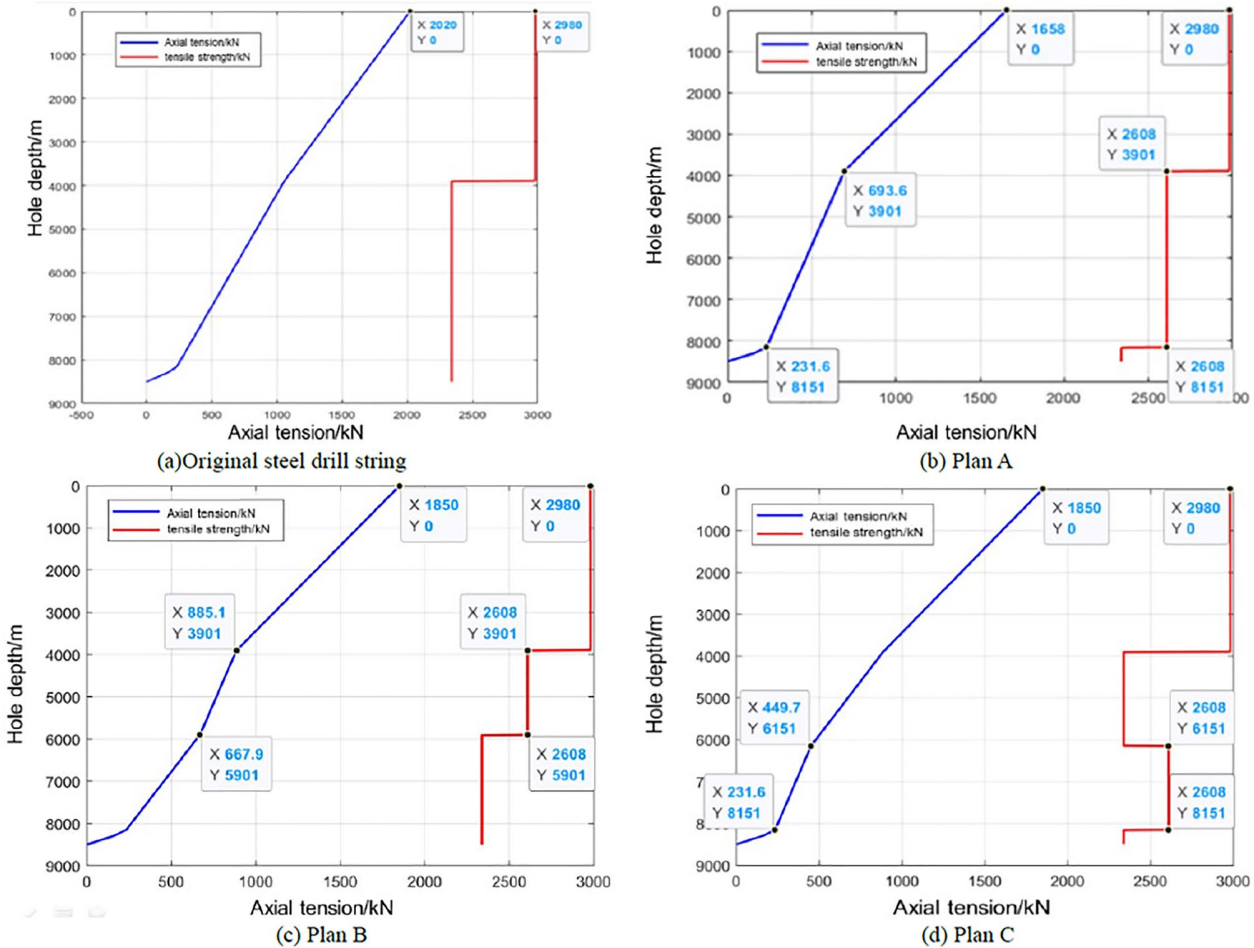
Axial force varies with well depth in original steel drill string (**a**) and three titanium/steel drill pipe composite drill string plans (**b**)–(**d**).

According to the static finite element model in “Finite element model” section, the static analysis was carried out on the original all-steel drill string and three titanium/steel drill pipe composite drill string plans. The maximum axial tensile force of the original steel drill string is 2020 kN and located at the wellhead, the tensile strength of the S135 steel drill pipe is 3311 kN. According to the safety factor of 0.9, the tensile allowance of the wellhead drill pipe is 960 kN, as shown in Fig. 6a. Under the same conditions, the maximum axial tensile force of the titanium/steel composite drill string plan A is 1658 kN and located at the wellhead. In Plan A, the tensile allowance of drill pipe at the wellhead is 1322 kN. The maximum axial force of the titanium drill pipe section is 693.6 kN, which is located at the top of the 4′′ drill pipe with well depth of 3900.7 m. Considering the tensile strength of the 4′′ titanium drill pipe is 2898 kN and the safety factor is 0.9, the tensile allowance of the titanium drill pipe is 1914.4 kN, as shown in Fig. 6b. For plans B and C, since the same length of the titanium drill pipe used, the maximum axial pulling force of the drill string at the wellhead is both 1850 kN, and the tensile allowance of drill pipe at the wellhead is both 1130 kN. For plans B and C, the maximum axial force of the titanium drill pipe section is 885.1 kN and 449.7 kN, respectively, which are located at well depths of 3900.7 m and 6151.0 m, respectively. Considering the same tensile strength of the 4′′ titanium drill pipe and the safety factor above, the tensile allowances of titanium drill pipes in plans B and C are 1722.9 kN and 2158.3 kN, respectively. It can be concluded from the above analysis that the tensile allowance of titanium/steel drill pipe composite drill string plans A, B and C at the wellhead are 1322 kN, 1130 kN and 1130 kN respectively, which are 362 kN, 170 kN and 170 kN higher than that of all-steel drill string respectively. The minimum tensile allowance of the titanium drill pipe section of plan C is 2158.3 kN, which is higher than 1914.4 kN and 1722.9 kN of plan A and B, indicating of higher safety, as shown in Fig. 6.

**Figure 7:**
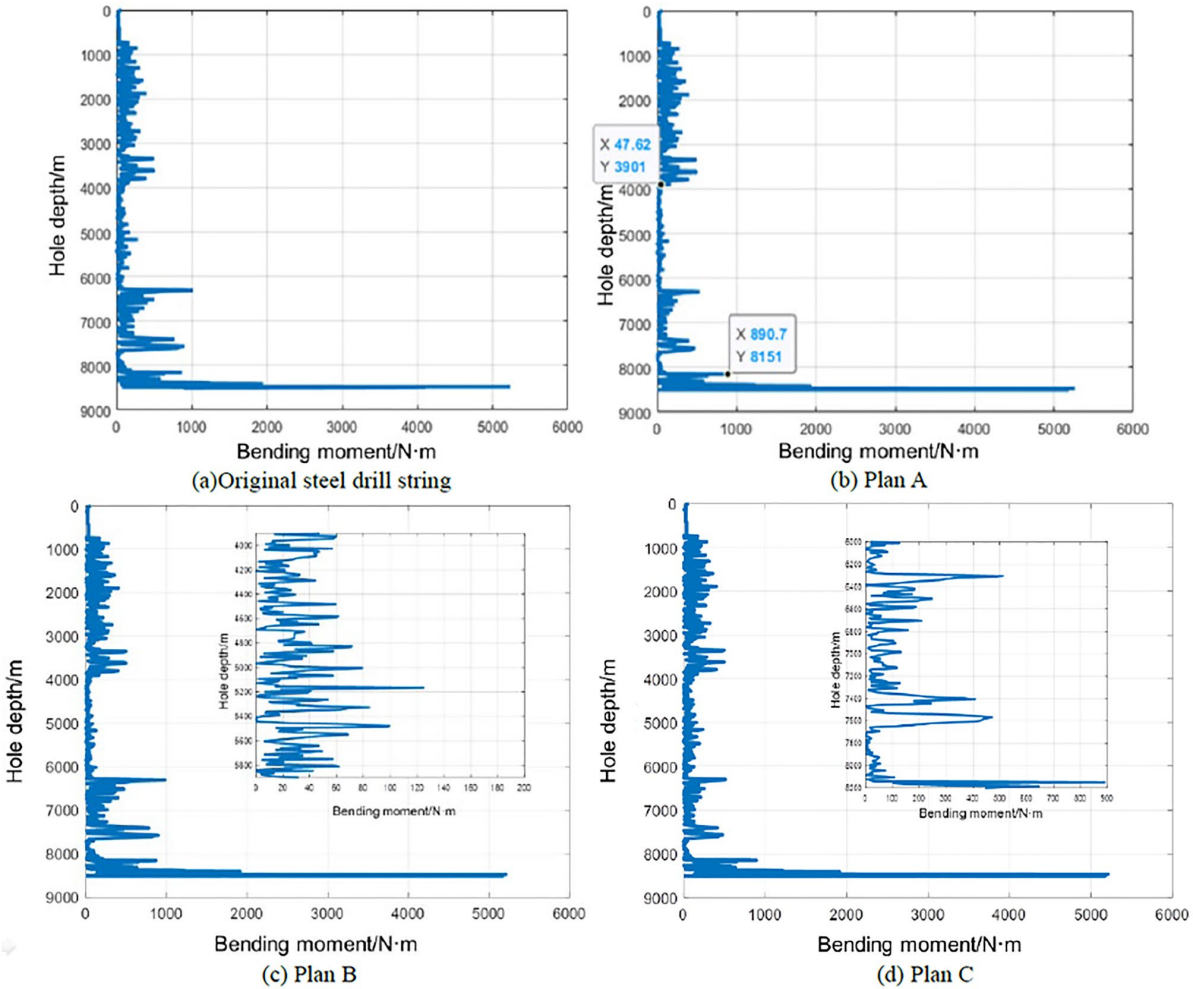
Bending moment varies with well depth in original steel drill string (**a**) and three titanium /steel drill pipe composite drill string plans (**b**)–(**d**).

Under the condition of the actual 3D wellbore trajectory, the bending moments of the four kinds of drill strings calculated according to the statics analysis of the whole drill string are shown in Fig. 7, it is revealed that the maximum bending moment of the original steel drill string is 5.23 kN m, which is located at a well depth of 8468.0 m. This is due to the relatively large curvature of the wellbore and the high rigidity of the drill collar. In other well sections, the bending moment values are all less than 1.0 kN m. In the three titanium/steel drill pipe composite drill string plans, the maximum bending moment of the titanium drill pipe section of Plan B is 0.13 kN m, which is less than 0.89 kN m of Schemes A and C, as shown in Fig. 7.

### Dynamic characteristics analysis

In the drilling process of ultra-deep wells, due to the influence of the eccentricity of the drill string, the excitation of the bit, the friction between the drill string and the wellbore, etc. the drill string will vibrate at a higher frequency, and displacement and dynamic stress are often greater than the results of statics. Especially the rotation of the drill string causes stress fluctuations in the curved section, and the vibration characteristics of the drill string can exacerbate the frequency of those stress fluctuations. Therefore, the calculation model of drill string based on static stress cannot reflect the influence of drill string vibration, and it is necessary to analyze the dynamic characteristics of drill string, especially the whirling characteristics of the drill string in the wellbore, including whirling trajectory and velocity^25^.

The dynamic characteristics of the drill string during the drilling process are very complex, closely related to the drill string structure, wellbore trajectory, operating parameters, etc. By comparing and analyzing the dynamic characteristics of different drill string structures under specific wellbore trajectories and operating parameters, a basis can be provided for the structural optimization design of titanium/steel composite drill strings. Based on the initial configuration of the static calculation of the drill string in the actual wellbore trajectory as described in “Static characteristics analysis” section, a rotational speed of 80 rpm is applied at the upper end of the drill string (i.e. the wellhead). A WOB of 60 kN is applied along the axis at the lower end of the drill string (i.e. at the drill bit), and the torque applied at the bit is determined by formula 30.

The boundary conditions of the drill string are: the upper end of the drill string (i.e. the wellhead) is at the wellhead, the drill string is hinged at the center of the wellbore, the axial direction is pulled by the hook load, and the torsion direction is the rotary speed. a specific rotational speed is applied in the torsion direction. The lateral motion at the lower end of the drill string (i.e. at the drill bit) is also a hinge boundary condition. The lower end is at the bit, and the lateral motion is also the hinge boundary condition, while the axial and torsional motions are respectively affected by the WOB and torque, respectively, and their magnitude depends on the interaction with the bit and the rock.

**Figure 8:**
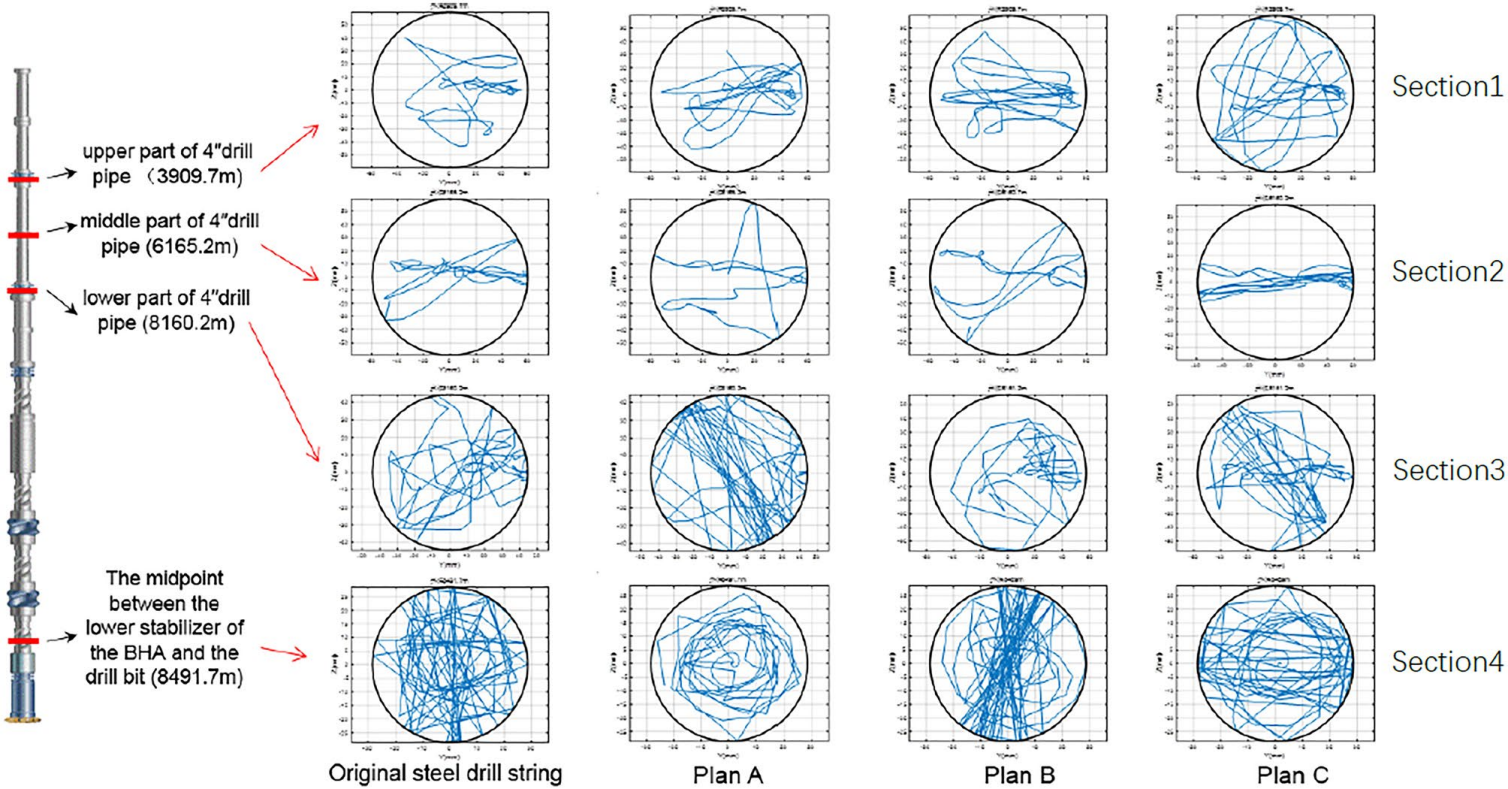
The whirling trajectory of original steel drill string and three titanium/steel drill pipe composite drill string plans at four points.

Considering factors such as the non-linear contact between the drill string and the wellbore, the finite element iterative method is used to calculate the whole drill string, and the whirling trajectory characteristics of each section of the drill string can be obtained. In order to compare the whirling trajectory of the original steel drill string and three titanium/steel drill pipe composite drill string plans, the whirling trajectories of four cross-sections as shown in Fig. 8 were analyzed: upper part of 4″drill pipe (well depth 3909.7 m, in “Introduction” section), middle part of 4″drill pipe (well depth 6165.2 m, in “Theoretical model” section), lower part of 4″drill pipe (well depth 8160.2 m, in “Structure design and mechanical characteristic analysis of titanium/steel drill pipe composite drill string” section) and the midpoint between the lower stabilizer of the BHA and the bit (well depth 8491.7 m, in “Dynamic safety evaluation of titanium/steel drill pipe composite drill string” section). It is revealed that the whirling trajectories of the four drill strings are similar at the “Introduction” and section “Theoretical model” sections. The friction and collision between the drill pipe and the wellbore are not frequent. Compared with the “Introduction” and “Theoretical model” sections, the whirling trajectories of the four drill strings show the characteristics of full wellbore movement at the “Structure design and mechanical characteristic analysis of titanium/steel drill pipe composite drill string” section, indicating that the collision contact between the lower section of the 4″ drill pipe and the wellbore is relatively frequent. Meanwhile, it can be seen from the whirling trajectory figure that the titanium drill pipe in plan A has more collisions with the wellbore at this position, even more than the original steel drill string, which may bring more possibility of collision damage. While the whirling trajectory of the other two titanium/steel drill pipe composite drill string plans is similar with the steel drill string. The whirling trajectories of the four kinds of drill string at the “Dynamic safety evaluation of titanium/steel drill pipe composite drill string” section show the characteristics of full wellbore movement, indicating that the collision and contact with the wellbore at this midpoint are relatively frequent.

The characteristics of normal collision between the original steel drill string and the wellbore are more obvious relatively, and the characteristics of the tangential rubbing between the titanium/steel drill pipe composite drill string and the wellbore are more obvious, it also can be found that the number of rubbings of plan A and plan C is significantly lower than that of plan B. Therefore, considering the economy of drilling operation, it can be concluded that the plan C of using titanium drill pipe for the lower 2000 m of the 4′′ drill pipe has the best whirling trajectory optimization effect by synthesizing the whirling trajectory characteristics of the whole drill string.

**Figure 9:**
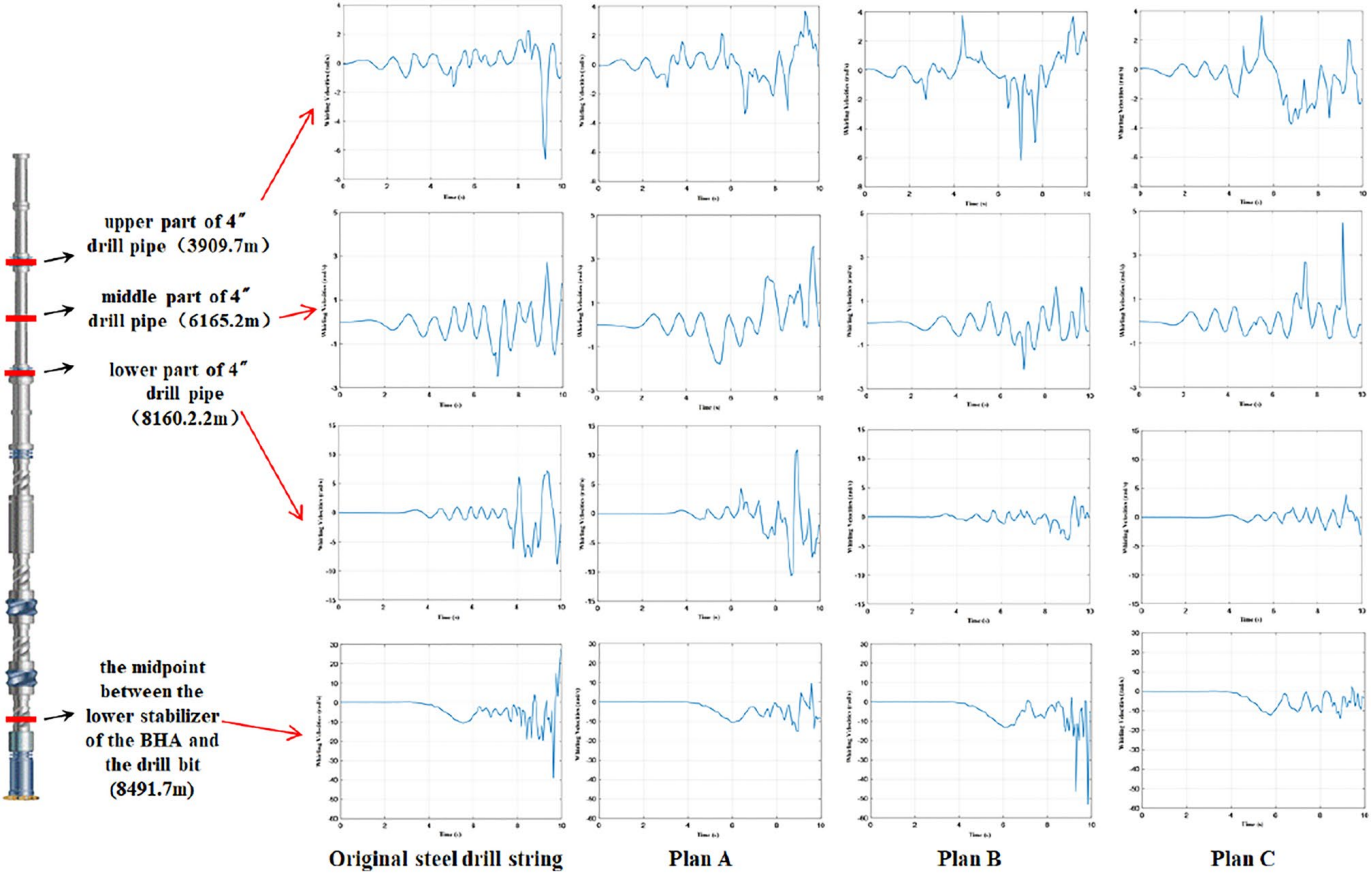
The whirling velocities of original steel drill string and three titanium/steel drill pipe composite drill string plans at four points.

Figure 9 shows the whirling velocity of original steel drill string and three titanium/steel drill pipe composite drill string plans at four points which are the same as the whirling trajectory analysis. It is can been seen that the whirling velocities of the four drill string structures at the upper part of 4″drill pipe (well depth 3909.7 m) and middle part of 4″drill pipe (well depth 6165.2 m) are all relatively small, and the fluctuation range of whirling velocities is close. However, at the position of lower part of 4″ drill pipe (well depth 8160.2 m), the whirling velocities of plan B and plan C are smaller than that of original steel drill string and plan A. Especially at the midpoint between the lower stabilizer of the BHA and the bit (well depth 8491.7 m), the whirling velocity of titanium/steel drill pipe composite drill string plan C is significantly lower than the other three drill strings, combined with the whirling trajectory analysis results, it is revealed that the titanium/steel drill pipe composite drill string of plan C has a low degree of lateral vibration and collision with the wellbore^26,27^.

**Figure 10:**
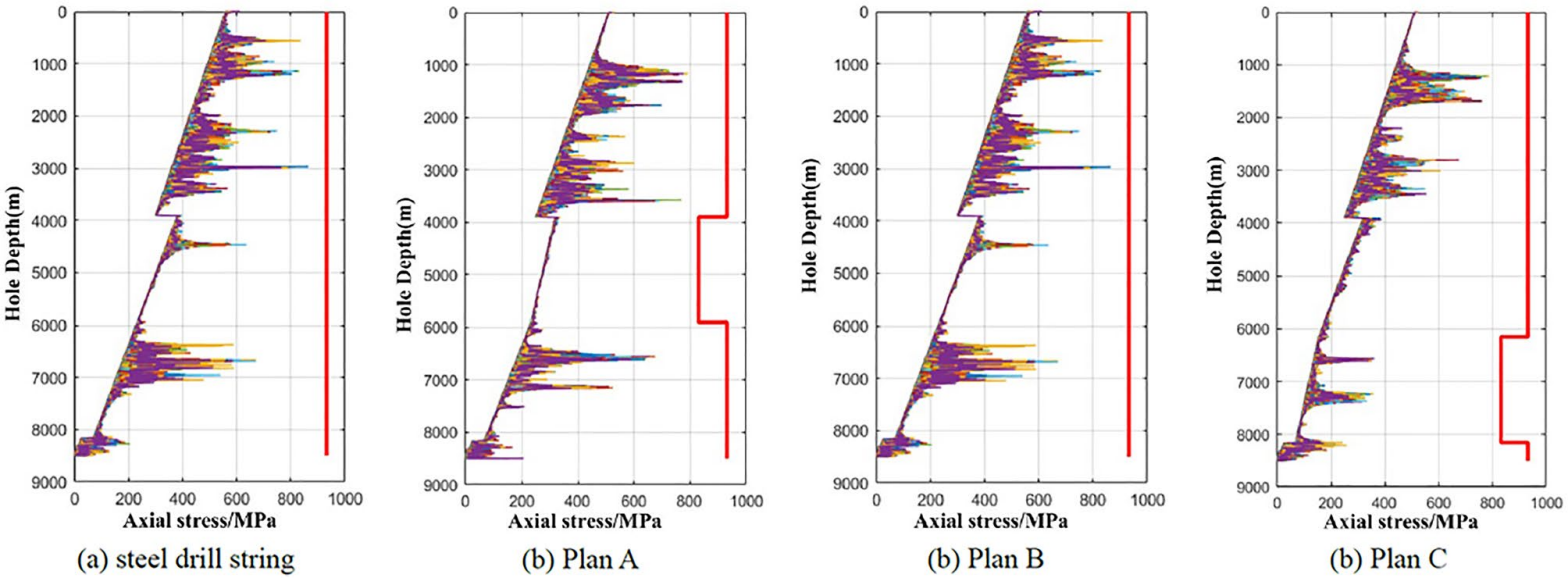
Composite diagram of dynamic axial stress and bending stress.

The main factor affecting the dynamic safety of the drill string in the dynamic analysis of the drill string is the dynamic stress of the drill string^28^. The greater the dynamic stress of the drill string, the smaller the dynamic safety factor of the drill string. Controlling and maintaining the dynamic stress of the drill string at a low level is the fundamental to ensure the safety of the drill string. Figure 10 shows the dynamic axial stress + bending stress variation curves of the original steel drill string and three titanium/steel drill pipe composite drill string plans respectively. It can be seen from the figure that the three titanium/steel drill pipe composite drill string all meet the material strength limit requirements. Due to the low density of titanium drill pipe, the dynamic stress level of the upper section of the titanium/steel drill pipe composite drill string is slightly lower than that of the all-steel drill string, and the dynamic stress level of the lower section of the drill string is significantly smaller than that of the all-steel drill string. It can be concluded that under the conditions of wellbore trajectory and drilling parameters described in this article, the dynamic stress level of the titanium/steel drill pipe composite drill string plan C is lower than that of the other two plans.

## Dynamic safety evaluation of titanium/steel drill pipe composite drill string

Due to factors such as the large slenderness ratio of the drill string, the complex structure of the wellbore, and the harsh geological environment, the drill string in ultra-deep wells can generate complex nonlinear vibrations, which affects the dynamic safety of the drill string. With the rapid increase in the number of deep and ultra-deep wells, the impact of drill string vibration on drilling safety and drilling efficiency is more prominent. Schlumberger has developed an independent quantified vibration risk technology based on seismological engineering theory^29^. It calculates a risk index with the collected vibration data, establishes the connection between the acceleration value and the parameters that cause failure and damage, and provides a quantitative method for controlling down-hole vibration. Based on the Schlumberger quantified vibration risk technology^30^, combined with drilling experience and data, this paper classifies the vibration shock risk of the titanium/steel drill pipe composite drill string, and evaluates the dynamic safety of the drill string. Through the statistics of the BHA acceleration peak, the impact risk is divided into three grades according to the impact number (Counts Per Second, CPS) of the acceleration peak exceeding 50 g (g is the acceleration of gravity, 9.8 m/s^2^), and is marked with green, yellow and red respectively, as shown in Table 2.

**Table 2:**
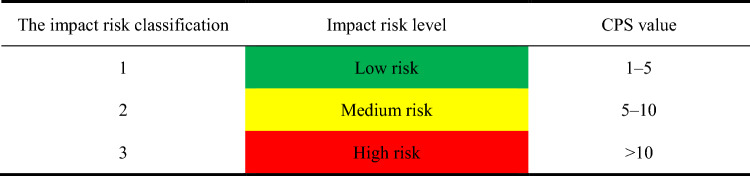
The impact risk classification.

**Figure 11:**
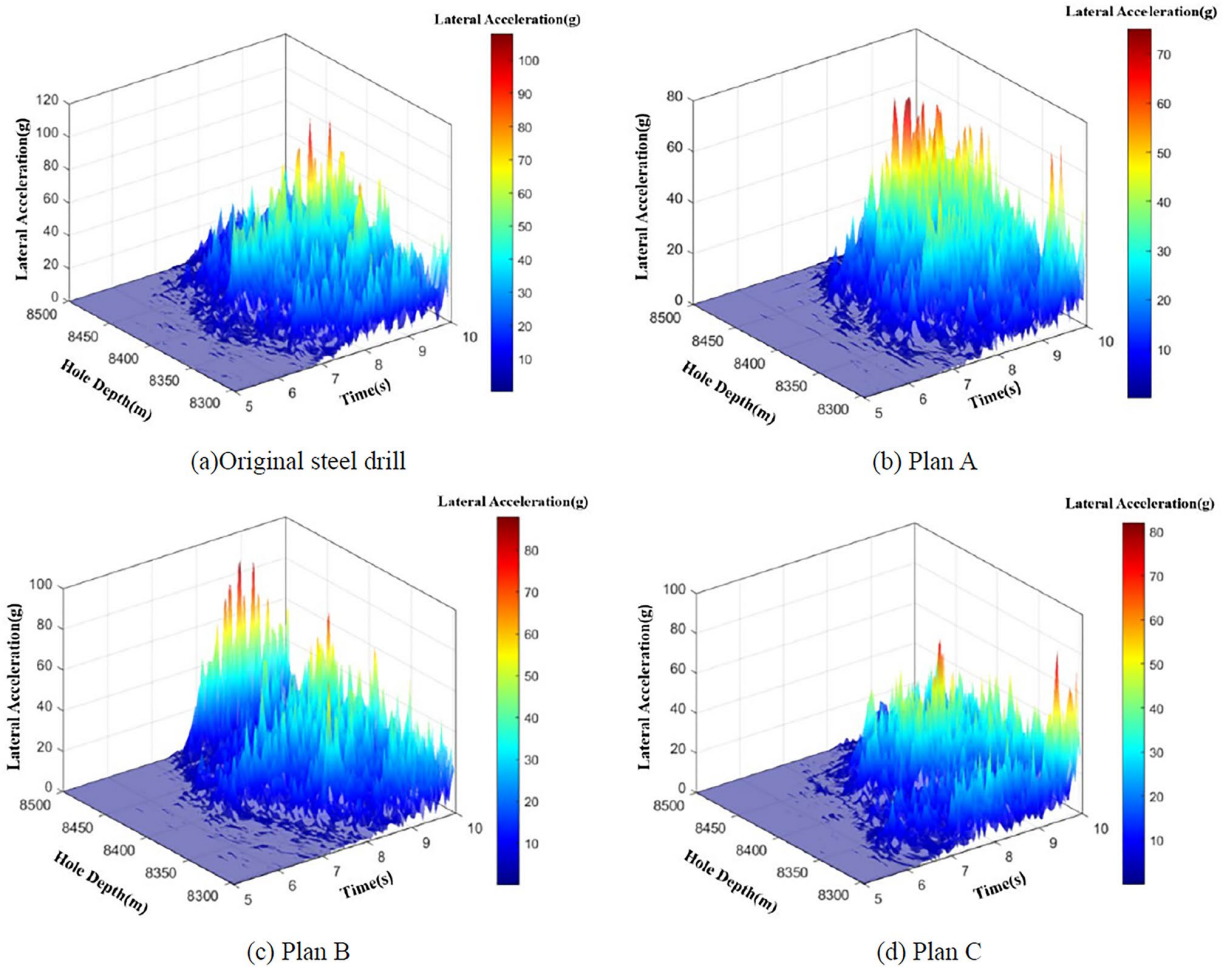
Vibration characteristics of different drill strings.

According to the dynamic analysis of the drilling string in ultra-deep wells, the number of the acceleration peak exceeding 50 g during the statistical time period (6–10 s) is counted, and then divided by the statistical duration (5 s) to obtain the CPS value of the drilling string. Thus, the impact risk level of the drill string can be evaluated based on this. Figure 11 show the vibration characteristics of different drill strings, the dynamic analysis results show that the maximum vibration acceleration of the original steel drill string is 107.8 g, CPS = 9; while the maximum vibration acceleration of the titanium/steel composite drill string plan A, B and C are 75.0 g, 87.9 g and 81.9 g, respectively, the CPS of the titanium/steel composite drill string plan A, B and C are 6, 3 and 4, respectively. According to the impact risk level shown in Table 1, the impact risk of the BHA part of the original steel drill string and the titanium/steel drill pipe composite drill string plan A are medium risk, and it can be seen from Fig. 11 that the vibration of the titanium/steel drill pipe composite drill is more gentle than that of the all-steel drill string, the impact risk of the BHA part of plan B and C are low risk. From the perspective of vibration characteristic strength, the vibration strength of the plan C is relatively small, so this paper will select the titanium/steel drill pipe composite drill string plan C to analyze and optimize its drilling operation parameters.

**Figure 12:**
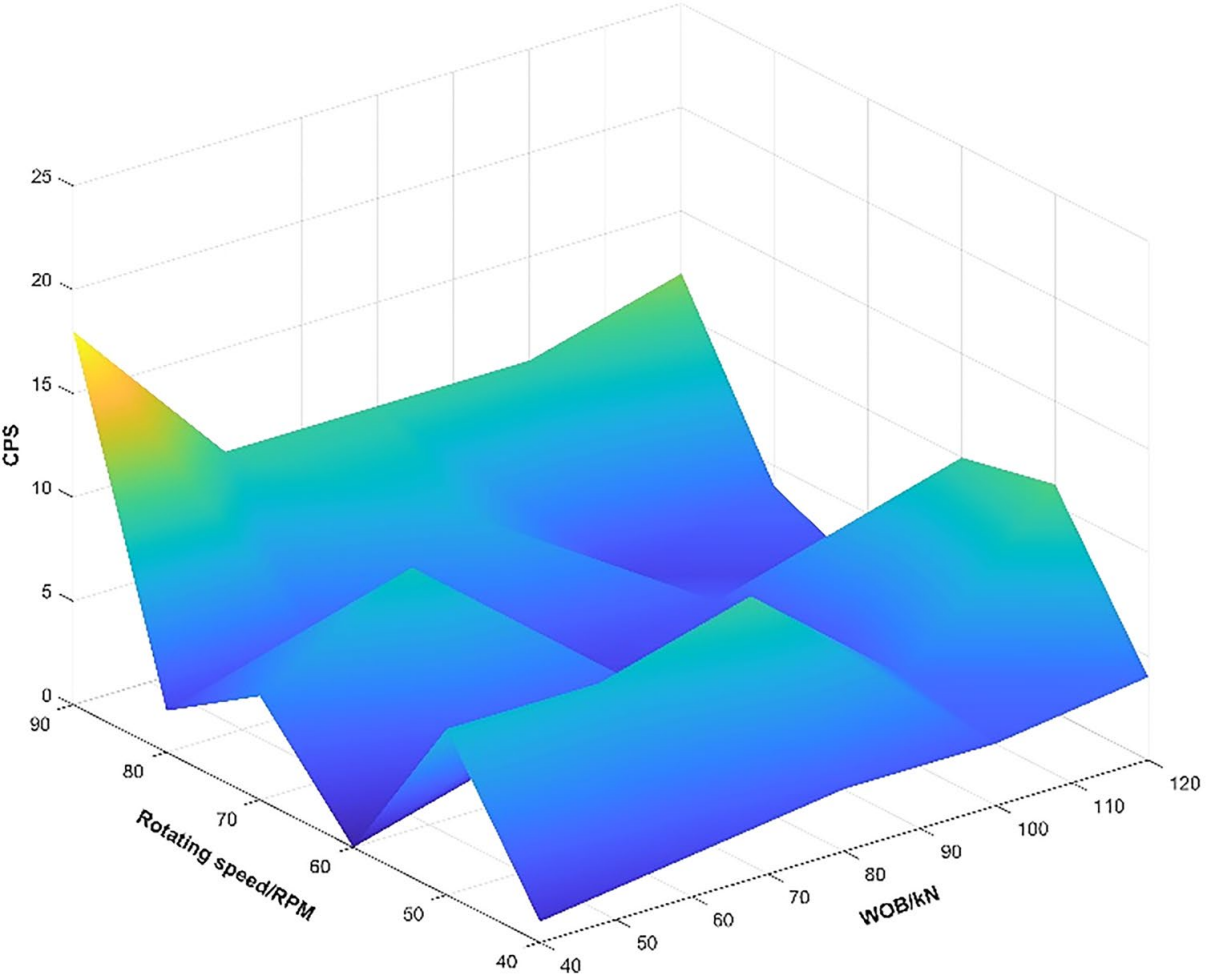
The relationship between WOB, rotational speed, and CPS value.

In order to provide recommended and safety operation parameters for the titanium/steel drill pipe composite drill string in drilling, taking the titanium/steel drill pipe composite drill string plan C as the target drill string, the vibration characteristics of the composite drill string under different drilling operation parameters (weight on bit(WOB): 40–120 kN; rotational speed 40–110 rpm) are analyzed. Combined with the impact level of the drill string shown in Table 3, the drill string vibration level was also classified, and the calculation results are shown in Table 4 and Fig. 12, it is revealed that when the rotational speed is 40 rpm, 70 rpm and 80 rpm, the impact times (CPS) of the titanium/steel drill pipe composite drill string with the acceleration peak exceeding 50 g at the BHA are less. At the same time, at higher rotational speed (70 rpm and 80 rpm), the acceleration peak and CPS are increased under the condition of medium and low WOB (40–80 kN). However, as the WOB and rotational speed increase, the chance of the titanium/steel drill pipe composite drill string being at high impact risk is greatly increased, especially under the conditions of 120 kN WOB and 50-60 rpm rotational speed, and under any WOB condition at 90 rpm rotational speed, the titanium/steel composite drill string are all at a high impact risk level, indicating that the drill string vibrates quite violently and has a high risk of failure.

**Table 3:** Calculated operating parameters of the titanium/steel composite drill string plan C.

Rotational speed	WOB/kN	Acceleration peak/g	CPS	Rotational speed	WOB/kN	Acceleration peak/g	CPS
40 rpm	40	50.5	1	50 rpm	40	97.7	8
	60	81.8	2		60	105.9	8
	80	75.1	3		80	109.3	10
	100	77.0	3		100	92.5	4
	120	82.8	4		120	92.0	11
60 rpm	40	49.9	0	70 rpm	40	86.1	5
	60	96.6	2		60	92.1	9
	80	83.8	3		80	86.0	2
	100	64.3	2		100	88.1	3
	120	92.0	10		120	79.6	2
80 rpm	40	65.3	2	90 rpm	40	140.5	18
	60	81.9	3		60	87.0	10
	80	80.5	7		80	104.6	10
	100	107.1	2		100	87.6	10
	120	94.0	4		120	102.0	12

**Table 4:**
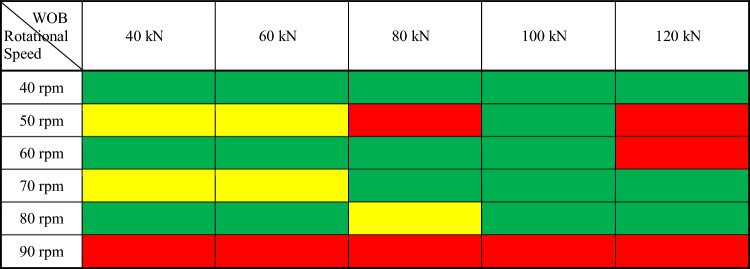
Optimal of operating parameters of the titanium/steel composite drill string plan C.

The calculated drill string vibration levels are graded and plotted according to the drill string shock risk levels in Table 3, the optimal of drilling operation parameters of the plan C was obtained and shown in Table 4. When using the titanium/steel drill pipe composite drill string structure of plan C for ultra-deep drilling, the combination of the three green drilling operation parameters in Table 4 should be preferred to ensure the high safety and reliability of the titanium/steel composite drill string.

## Conclusions

In the present study, the static and dynamic characteristics of different titanium/steel drill pipe composite drill string under drilling conditions was investigated, and the dynamic model is solved by the node iteration model and the Newmark-βmethod, the dynamic characteristics of three titanium/steel composite drill strings were analyzed and optimized by using whirling characteristics, dynamic stress and vibration acceleration, the following conclusions could be drawn in this work:

1. Based on the drilling conditions of the ultra-deep well, the static and dynamic model of the titanium/steel drill pipe composite drill string were established according to the actually measured wellbore trajectory, the node iteration method is used to judge whether contact occurs, and the Newmark-βmethod is used to calculate the spatial configuration of the drill string. Due to the small rigidity of titanium drill pipe, the bending moment of the titanium/steel drill pipe composite drill string in the actual wellbore trajectory is smaller than that of the original steel drill string.
2. As the density of titanium alloy is only 56% of that of steel, the tensile allowance of the wellhead is greatly increased after using titanium drill pipe. The static finite element analysis results indicate that the tensile allowance of titanium/steel drill pipe composite drill string plans A, B and C at the wellhead are 1322 kN, 1130 kN and 1130 kN respectively, which are 362 kN, 170kN and 170 kN higher than that of all-steel drill string respectively.
3. The elastic modulus of titanium drill pipe is only 52% of that of steel drill pipe. The results of dynamic analysis show that the use of titanium drill pipe can significantly reduce the whirling velocity, dynamic stress and vibration acceleration of drill string due to its small stiffness. At the same time, the three titanium/steel drill pipe composite drill string plans indicate that the position of the titanium drill pipes has a significant impact on the dynamic characteristics of the drill string.
4. According to the statistics of the BHA acceleration peak, the impact risk of the drill string vibration is divided into three grades according to the impact number (CPS). Based on the dynamic characteristics of drill string under different WOB and rotational speed, the drilling operation parameter optimization method for titanium/steel drill pipe composite drill string has been developed. These high-risk drilling operation parameters should be avoided in the drilling of ultra-deep well.
5. Considering the factors of safety and cost, three titanium/steel drill pipe composite drill string were designed according to the different installation positions and lengths of the 4″titanium drill pipe in the drill string. The plan C of using titanium drill pipe for the lower 2000 m of the 4′′ drill pipe has the lower dynamic stress level, a small degree of lateral vibration, and a low degree of collision with the wellbore.
